# Tuberculosis of the Heart: A Diagnostic Challenge

**DOI:** 10.3390/tomography8040137

**Published:** 2022-06-22

**Authors:** Karuna M. Das, Taleb Al Mansoori, Yousef Habeeb Alattar, Klaus V. Gorkom, Ali Shamisi, Anisha Pulinchani Melethil, Jamal Aldeen Alkoteesh

**Affiliations:** 1 College of Medicine and Health Sciences, United Arab Emirates University, Sheikh Khalifa Bin Zayed Street, Asharej, Al Ain P.O. Box 17666, United Arab Emirates; taleb.almansoor@uaeu.ac.ae (T.A.M.); klausg@uaeu.ac.ae (K.V.G.); 2 Sheikh Khalifa Medical City, Abu Dhabi P.O. Box 51900, United Arab Emirates; yalattar@seha.ae; 3 Tawam Hospital, Al Maqam, Tawam, Abu Dhabi P.O. Box 15258, United Arab Emirates; arshamisi@seha.ae (A.S.); jkoteesh@seha.ae (J.A.A.); 4 Department of Data Science, MAHE, Manipal 576104, Karnataka, India; anishastat@gmail.com

**Keywords:** tuberculous myopericarditis, magnetic resonance imaging, transmural mesocardial and epicardial fat late enhancement, irregular thickening of pericardium

## Abstract

Tuberculosis of the heart is relatively rare and presents a significant diagnostic difficulty for physicians. It is the leading cause of death from infectious illness. It is one of the top 10 leading causes of death worldwide, with a disproportionate impact in low- and middle-income nations. The radiologist plays a pivotal role as CMR is a non-invasive radiological method that can aid in identifying potential overlap and differential diagnosis between tuberculosis, mass lesions, pericarditis, and myocarditis. Regardless of similarities or overlap in observations, the combination of clinical and certain particular radiological features, which are also detected by comparison to earlier and follow-up CMR scans, may aid in the differential diagnosis. CMR offers a significant advantage over echocardiography for detecting, characterizing, and assessing cardiovascular abnormalities. In conjunction with clinical presentation, knowledge of LGE, feature tracking, and parametric imaging in CMR may help in the early detection of tuberculous myopericarditis and serve as a surrogate for endomyocardial biopsy resulting in a quicker diagnosis and therapy. This article aims to explain the current state of cardiac tuberculosis, the diagnostic utility of CMR in tuberculosis (TB) patients, and offer an overview of the various imaging and laboratory procedures used to detect cardiac tuberculosis.

## 1. Introduction

Tuberculosis of the cardiovascular system is pretty rare. Even though tuberculosis is generally treatable, it continues to be a primary cause of illness and death globally, ranking second only to HIV as the most prevalent infectious disease [1]. Cardiovascular tuberculosis has had a remarkable resurgence in the context of the HIV epidemic, with increased extrapulmonary or diffuse involvement. TB accounts for virtually all pericardial effusions among HIV-positive patients in Sub-Saharan Africa, compared to 50–70% in the general population and fewer than 5% in the industrialized world [2]. Tuberculous involvement heart occurs in up to 0.3 percent of autopsied patients [3]. Pericardial tuberculosis (TB) is found in about 1% of all TB autopsied cases and in 1% to 2% of cases of pulmonary TB [4]. Isolated tuberculous myopericarditis is extremely rare; however, it is more common in HIV-positive people and those on chronic immunosuppression, with a 51% prevalence rate [2]. CMR has a significant advantage over echocardiography for detecting, characterizing, and assessing cardiovascular abnormalities. This article aims to provide an overview of the present state of cardiac tuberculosis, the diagnostic value of CMR in tuberculosis patients, and the various imaging and laboratory tests utilized to detect cardiac tuberculosis.

## 2. Pathogenesis of Tuberculosis of the Heart

Myocardial tuberculosis, first diagnosed by Maurocordat on autopsy in 1664, is unusual and often goes undiscovered while the patient is still alive [5]. Cardiopulmonary tuberculosis accounts for between 1% and 2% of all tuberculosis cases in immunocompetent persons [6]. In a study of 19 individuals with cardiovascular TB, one case was diagnosed antemortem, 11 had nodular lesions on autopsy, and 7 had miliary lesions; only one patient had acid-fast bacilli [7]. TB infection of the cardiovascular system is transmitted mostly retrogradely from mediastinal lymph nodes, hematogenously from a primary tuberculosis infection, and rarely contiguously [8,9]. Several studies have found that the right ventricle and right atrium are the most typically impacted, most likely due to the frequent involvement of right mediastinal lymph nodes and subsequent myocardial involvement. [7,10,11,12]. A Tuberculous pericarditis has four recognized pathological stages: (1) fibrinous exudation with initial polymorphonuclear leukocytosis with the loose organization of macrophages and T cells; (2) serosanguineous effusion with a predominantly lymphocytic exudate composed of monocytes and foam cells; (3) effusion absorption with the organization of granulomatous caseation and pericardial thickening; and (4) constrictive scarring [8].

## 3. Clinical Presentation of Tuberculosis of Heart

TB of the myocardium may manifest itself in many ways. Acute pericarditis is a rare clinical presentation of tuberculous pericarditis, accounting for only 3% to 8% of patients who present with tuberculous pericarditis. It is characterized by severe pericarditic chest pain, pericardial friction rub, widespread ST-segment and T wave abnormalities, and PR segment depression [13,14]. On the other hand, the presentation is more subtle, and systemic signs and symptoms, such as chest pain, fever, dyspnea, pericardial rub, paradoxical pulse, and hepatomegaly are more prevalent [15]. Clinical symptoms are influenced by the rate of fluid accumulation, the hemodynamic effect on cardiac contraction, and the degree of infection-induced inflammation [16]. The most common symptoms of fluid accumulation are broad systemic symptoms or heart failure [17,18]. When fluid accumulates rapidly, and compensatory mechanisms are unavailable, the patient develops tachycardia and hypotension. With inadequate treatment, up to half of patients may develop cardiac tamponade, with mortality rates as high as 85% at six months [19,20]. Symptoms of acute tuberculous myocarditis include abnormalities in the conduction system, such as prolonged QT syndrome, ventricular fibrillation, or cardiac arrest [6,8,21,22,23,24,25]. On the other hand, chronic myocardial involvement might emerge as increasing heart failure symptoms or as an asymptomatic patient discovered postmortem [26]. Subacute endocarditis presents with similar symptoms as pericarditis. Symptoms such as dyspnea and heart failure emerge when vegetation affects hemodynamics and severely decreases valve function [27]. Additionally, the literature has identified the blockage of the right ventricle’s outflow tract, ventricular aneurysm, ventricular pseudoaneurysm, aortic insufficiency, valvular endocarditis, myopericarditis, coronary arteritis, congestive heart failure, valvular endocarditis, and myopericarditis [6,8,21,22,23,24,25,28].

## 4. Laboratory Diagnostic Test

To achieve a conclusive diagnosis of cardiac tuberculosis, the tubercle bacillus must be isolated directly or via culture from pericardial fluid, the heart, or vascular tissue; however, isolation can be challenging in practice. Lopez et al. developed an approach for identifying tuberculosis in the cardiovascular system by combining clinical, laboratory, and radiological examinations, including cardiac CT and CMR [9]. Pericardiocentesis was suggested in such situations where pericardial effusion was present, along with clinical indications of night sweats, weight loss, and fever [9]. Numerous tests may be employed to confirm infection, each having its own diagnostic performance. The test should be chosen based on the likely location of cardiovascular impairment and the availability of the test in the local environment. Clinical examination and specific pericardial fluid tests for adenosine deaminase (ADA) levels, gamma interferon (IFN-y), and polymerase chain reaction testing for Mycobacterium tuberculosis are all used to diagnose tuberculosis [20]. Mutyaba et al. recommend the following diagnostic method for TB pericarditis in endemic locations in their proposal: (1) Obtain biomarkers such as IFN-y or ADA levels in pericardial fluid and confirm tuberculosis in other places to rule out other potentially fatal causes of an inflammatory exudative effusion (e.g., bacterial, neoplastic, or uremic) (e.g., lymph nodes, sputum, or pleural fluid) [8,29]. The enzyme ADA is required for the conversion of adenosine to inosine. It can be present in many tissues, although lymphoid tissue has the highest concentration. Increased ADA activity seems to be linked to an activated lymphocyte antigenic response in tuberculosis [8]. In a systematic review and meta-analysis, ADA testing had a sensitivity and specificity of 88% and 83%, respectively, with an area under the receiver operating characteristic curve of 0.9539 [9]. Thus, ADA activity has a significant diagnostic value for pericardial tuberculosis [8]. Furthermore, in pericardial fluid, unstimulated IFN-y is a TB biomarker. Its value is based on the fact that T lymphocytes create IFN-y when they are exposed to specific antigens [30]. Compared to the criterion standard, a cut-off of 50 pg/mL resulted in 92% and 100% sensitivity and specificity [9]. Despite its high diagnostic accuracy, the test is not commonly used because of its high cost, especially in low- and middle-income nations. The (PCR, polymerase chain reaction) test, is a molecular biology technique for duplicating a particular sequence of DNA (Deoxyribonucleic acid). Although the polymerase chain reaction method has a high specificity (between 96% and 100%) for detecting tuberculosis in pericardial fluid, some studies have shown that it has a low sensitivity (15–20%) [20,31]. When the three pericardial TB tests were compared, IFN-y had the best sensitivity and specificity, with 95.7% sensitivity and 96.3% specificity, suggesting that IFN-y is the most effective diagnostic test. Laboratory testing including PCR is a molecular biology technique that is currently the quickest and most sensitive when applied to pericardial fluids; nonetheless, liquids culture for TB remains the GOLD STANDARD for laboratory diagnosis.

## 5. Role of Cardiac MRI

### 5.1. TB pericarditis and Myopericarditis

Myopericarditis is a term used to describe a primarily pericarditic condition. Pericarditis is usually linked with myocarditis of varying severity. Due to their similar causal agents, cardiotropic viruses, pericarditis, and myocarditis may coexist in clinical practice [28]. The evaluation of pericardial disorders requires more than morphologic examination; the diagnostic challenge is to ascertain the effect of the abnormal pericardium on cardiac filling. The ability to combine anatomic and functional data in a single exam, the ability to characterize tissue and determine the presence and severity of inflammation and disease activity, and the value of CMR in accurately assessing the rest of the heart, particularly the myocardium, which is currently a diagnostic challenge, are all compelling arguments in favor of CMR.

**Figure 1:**
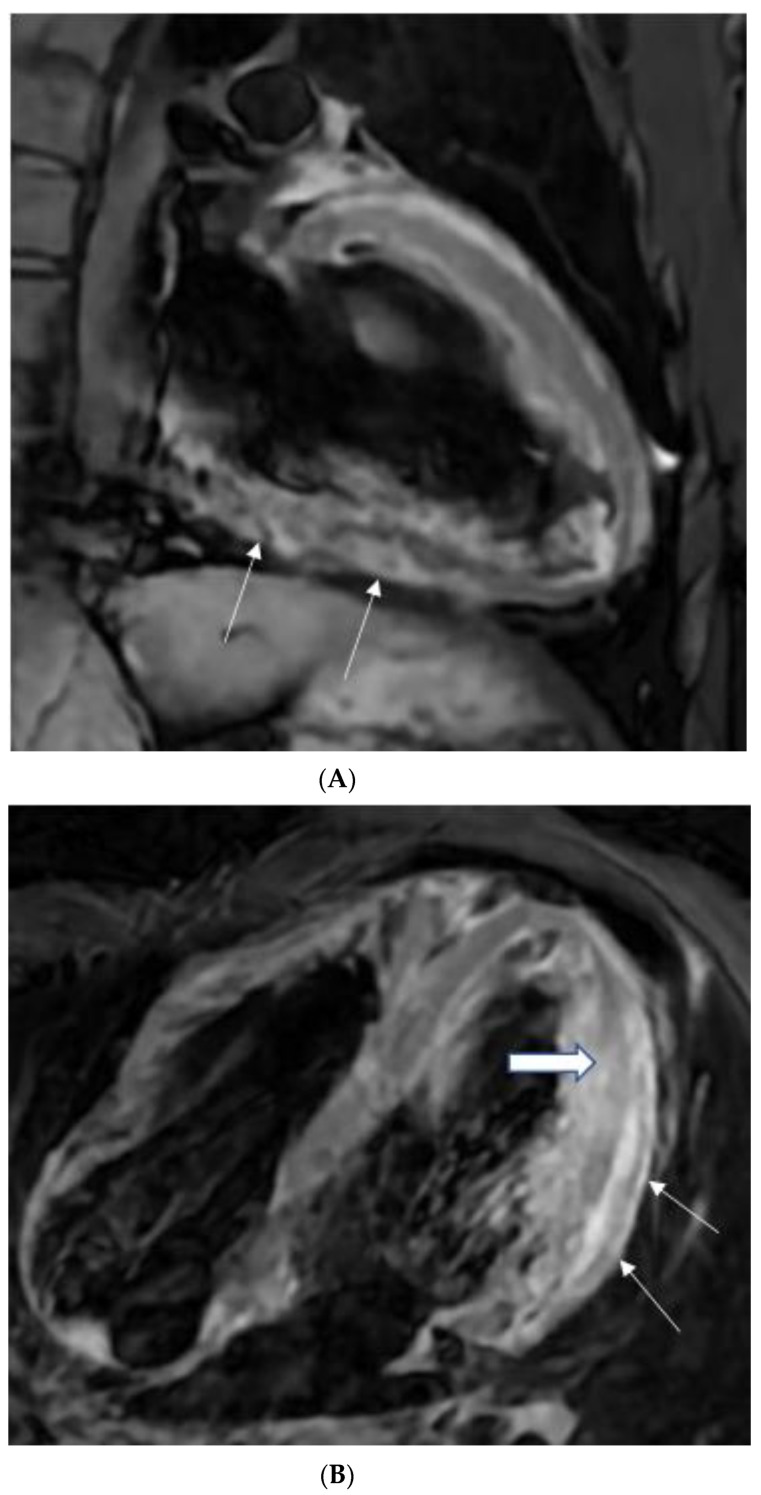
(**A**) A 35-year-old male with a recent history of treated tuberculous pericarditis for six months had CMR for ventricular tachycardia. 2CH T2w STIR pictures demonstrate increased epicardial and transmural signals in the inferior wall (arrows). (**B**) A 35-year-old male with a recent history of treated tuberculous pericarditis for six months had CMR for ventricular tachycardia. 4CH T2w STIR pictures demonstrate irregular thickening of the pericardium (small arrows) with the increased transmural signal of the mid-lateral wall (thick arrow).

**Figure 2:**
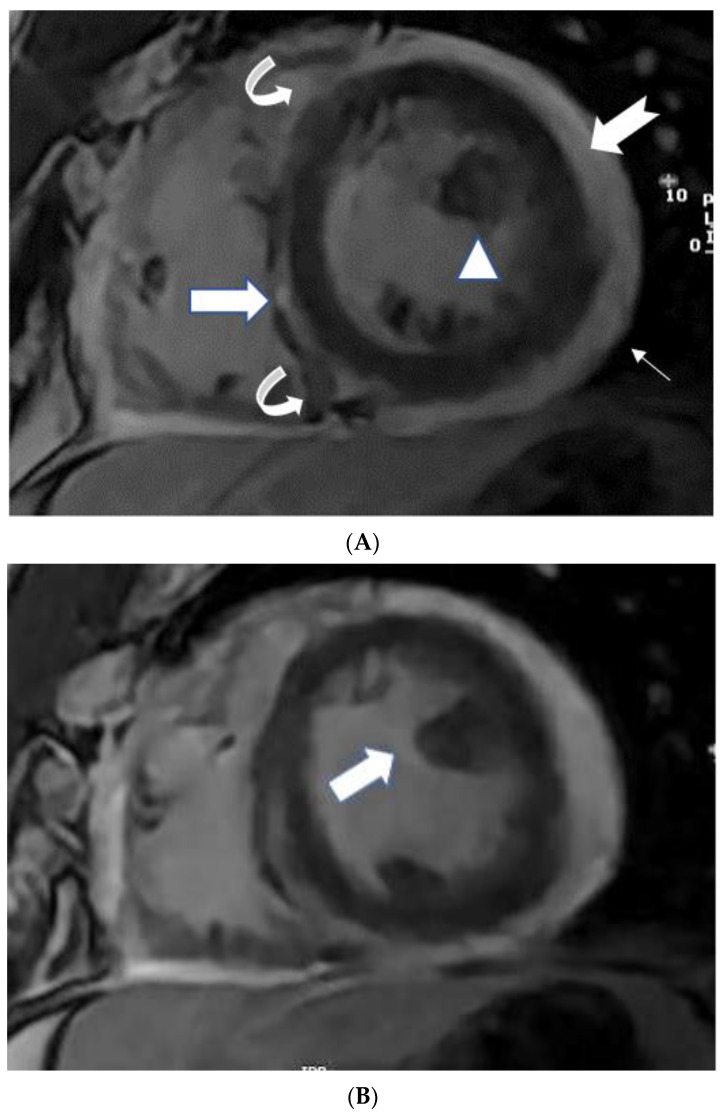

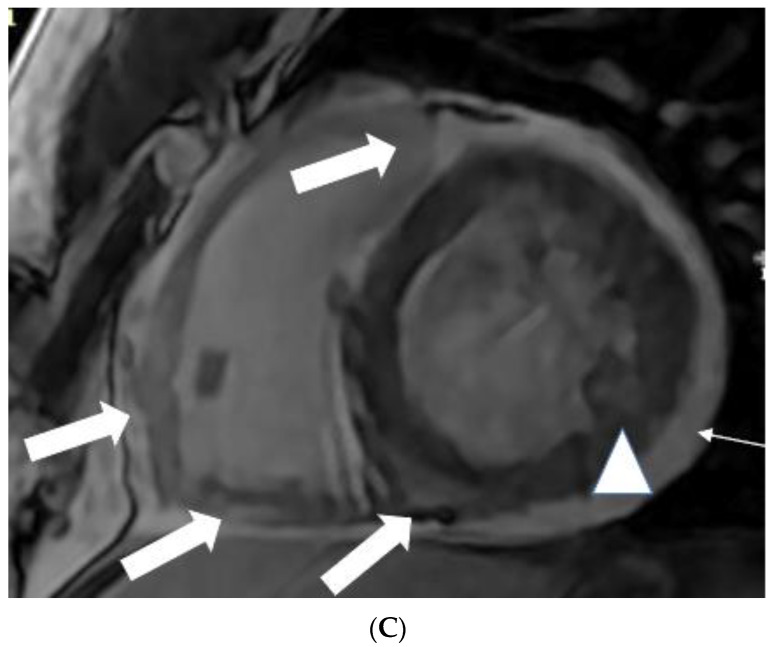
(**A**) A 35-year-old male with a recent history of treated tuberculous pericarditis for six months had CMR for ventricular tachycardia. Short axis mid myocardial slice, late enhancement images, shows subepicardial anterolateral (notched arrow), mesocardial mid and inferior septal (white arrow), anterior and posterior RV insertion points (curve arrow) with thickened pericardium and epicardial fat enhancement (thin arrows). Enlarged AL papillary muscle (arrowhead). (**B**) A 35-year-old male with a recent history of treated tuberculous pericarditis for six months had CMR for ventricular tachycardia. Short axis mid myocardial slice late enhancement images show all the features of (**A**) and asymmetrical enlargement of the AL papillary muscle (arrow). (**C**) A 35-year-old male with a recent history of treated tuberculous pericarditis for six months had CMR for ventricular tachycardia. Short axis mid myocardial slice late enhancement images show the RV lateral and inferior wall (arrow), superior and inferior insertion (white arrow) with epicardial fat enhancement (thin arrows). PM papillary muscle is with central enhancement (arrowhead).

**Figure 3:**
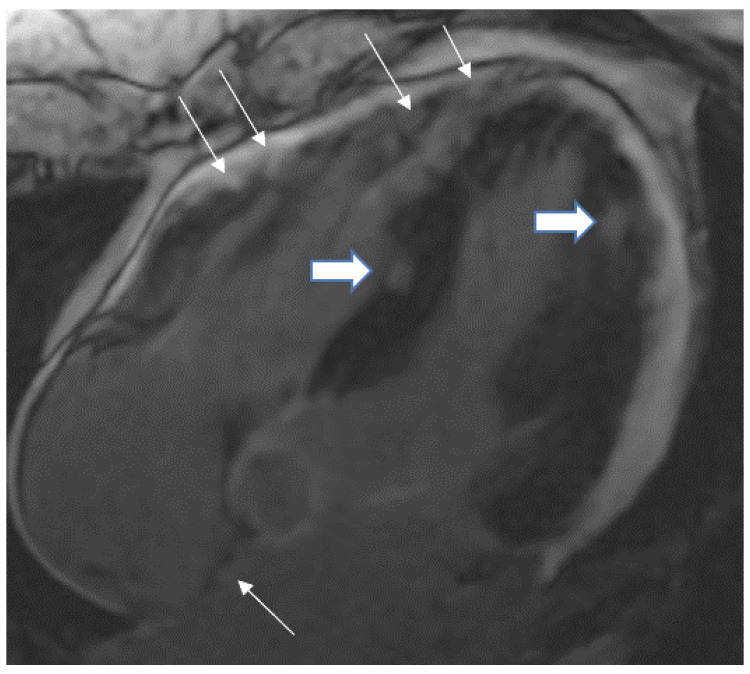
A 35-year-old male with a recent history of treated tuberculous pericarditis for six months had CMR for ventricular tachycardia. 4 CH, late enhancement images, shows RV free wall (epicardial and transmural thin arrows), inferior septal (transmural- thick Arrow), lateral wall LV (transmural-thick arrow), and interatrial septum (thin arrow) enhancement.

Detecting inflammatory pericarditis has become easier with the development of new CMR techniques [32,33]. T1-weighted spin-echo CMR or cine CMR can detect pericardial layer thickness and simultaneous pericardial effusion, whereas T2-weighted STIR spin-echo CMR can detect pericardial layer edema. Pericardial enhancement in gadolinium-enhanced CMR investigations is an appropriate approach for detecting pericardial inflammation (Figure 1, Figure 2 and Figure 3). Both LGE CMR and spin-echo CMR are advantageous. A fat-suppressive prepulse could enhance the visibility of pericardial inflammation [32]. Delineation of the pericardial layer, which may become more irregular in chronic pericarditis, and streaky enhancement of the surrounding fat and adjacent myocardial tissue, indicate concomitant epicarditis or myocarditis and are additional intriguing imaging characteristics [32]. Yelgec and colleagues recently discovered pericardial enhancement in 9 persons and pericardial effusion in 6 of 20 patients tested with CMR for clinical suspicion of acute myocarditis, confirming the prevalence of myopericarditis [34]. Furthermore, Sayed et al. observed that 53.1% of patients with tuberculous pericarditis with pericardial enhancement developed myopericarditis, as evidenced by reduced left ventricular systolic function or higher circulating myocardial enzymes, in their first prospective investigation on cardiovascular TB [2]. Hence, both results demonstrated that myocarditis and pericarditis may be produced by the same etiologic factor and coexist in the same patient.

Pericardial constriction is most often idiopathic, although it may also be caused by inflammatory pericarditis, such as TB. Tuberculosis has declined compared to other cardiac surgery-related illnesses, whereas radiation-induced pericarditis has increased [35]. High systemic venous pressures and inadequate cardiac output are common signs of pericardial constriction [36]. Chronic fibrosing pericarditis is characterized by a thicker, fibrotic, and calcified pericardium that constricts the heart and affects cardiac filling [37]. Therefore, the diagnosis of right heart failure should always be checked when patients present with symptoms [38]. Equalizing end-diastolic pressures in all four cardiac chambers and enhanced ventricular coupling are pathophysiological features of pericardial constriction caused by the confinement of the cardiac chambers by the stiff, constant pericardial volume [35,39].

The thickness of the pericardium has long been recognized as a strong predictor of constrictive pericarditis [40,41]. The thickness of the pericardium varied between 1.5 and 3.9 mm in several locations with tuberculous myopericarditis (Figure 1B). On pathologic examination, the maximum pericardial thickness varies significantly (1–17 mm; mean, 4 mm) in patients with pericardial constriction (96%), with up to 20% of patients having a regular (2-mm) thickness [40,41]. According to Feng et al., the pericardial thickness was significantly lower in individuals with chronic constrictive pericarditis than in those with reversible constrictive pericarditis (2 mm ± 1 vs. 4 mm ± 1; *p* > 0.001) [42]. Thus, patients with end-stage constrictive pericarditis had a narrower pericardium than those with chronic inflammation that survived. In addition, CMR tagging techniques may be advantageous for precisely displaying fibrotic pericardial adhesions [32].

### 5.2. TB Myocarditis

**Figure 4:**
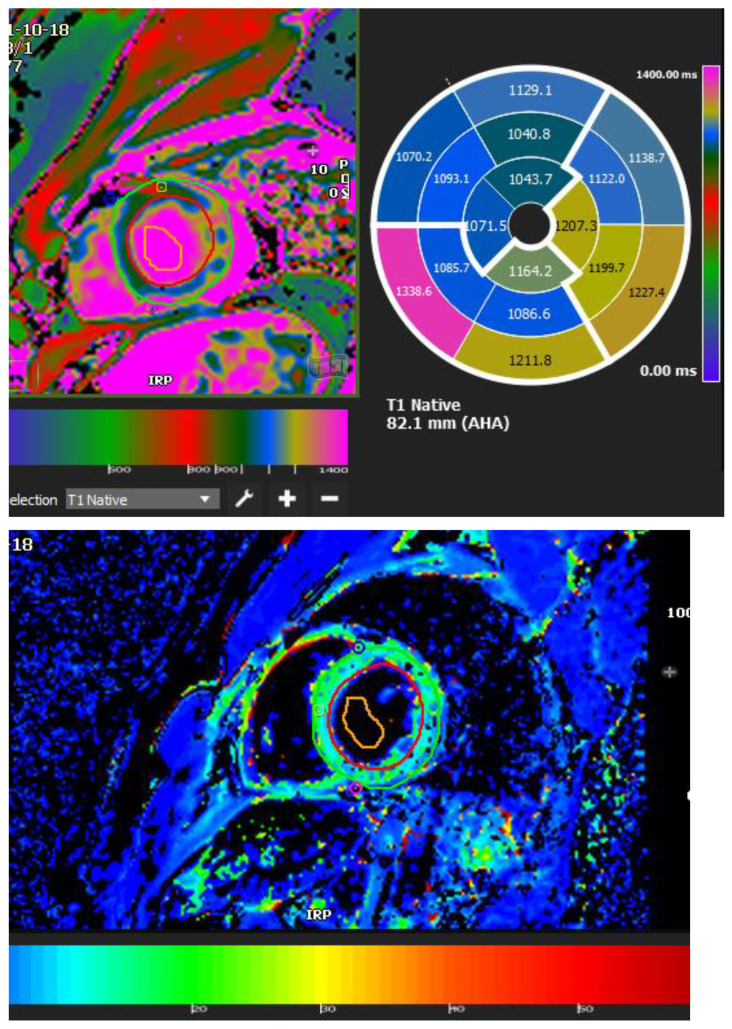
A 35-year-old male with a recent history of treated tuberculous pericarditis for six months had CMR for ventricular tachycardia. A native T1 map of 1132 ± 178 (Figure 4) and ECV of 25 ± 8%.

Myocarditis is assumed to involve both ventricles diffusely or, less commonly, the right ventricle alone. In one of the studies with endomyocardial biopsy, biventricular involvement was noted in approximately 70% of patients and alone RV myocarditis in 8% [43]. Myocardial involvement may be identified by aberrant ECG changes, transient localized and global wall motion abnormalities, and increased cardiac enzyme levels. The CMR images of the patient included here (Figure 1, Figure 2, Figure 3 and Figure 4) presented with VT and elevated cardiac enzymes with a clinical diagnosis of myopericarditis. CMR verified all three Lake Louise Consensus Criteria and decreased biventricular ventricular function in the present case. There was a disproportionate LGE in the RV free lateral wall, inferior wall, superior and inferior RV/LV junction, and interatrial septum (Figure 2). It supports the etiopathogenesis of cardiac TB by indicating that the right side is more frequently impacted, most likely due to the proximity of the involved right mediastinal lymph node [7,10,11,12]. Patchy transmural, mesocardial (septal), and epicardial LGE are also seen in the left ventricle (Figure 2A). The regional wall motion abnormality of the inferior septum (Figure 2A) corresponds well with the mesocardial enhancement, and it may represent an area of active inflammation evolving into a chronic scar due to tuberculous infiltration [44]. The accuracy of diagnosing myocarditis was further substantiated by a native T1 map of 1132 ± 178 (Figure 4) and ECV of 25 ± 8%. People with acute chest discomfort may be distinguished from those with acute coronary syndrome or myocarditis using the native T1 and ECV [45]. For identifying and assessing diffuse myocardial fibrosis and edema, native T1 and ECV mapping are more sensitive than LGE [45].

### 5.3. Papillary Muscle Enlargement

Enlargement of the papillary muscles has not been described before in tuberculous myopericarditis (Figure 2B). Hypertrophy or infiltrative illnesses (e.g., amyloidosis, sarcoidosis, iron deposition) may thicken papillary muscles, affecting papillary muscles [44]. Although coronary atherosclerosis is the most common cause of LGE of the papillary muscle without rupture, this disorder can also be caused by shock, infectious endocarditis, acute valvular regurgitation, anemia, LVOT blockage, systemic hypertension, cardiomyopathies, endocardial fibroelastosis, endomyocardial fibrosis, and myocardial diseases [44,46].

### 5.4. Intracardiac Tuberculoma

Tuberculomas are mass-like lesions of tuberculosis of the heart [5,47,48]. Endocardial tuberculoma is exceedingly uncommon, occurring in just 19 of 13,658 autopsy cases (0.14%) [7]. Myocardial tuberculomas are more often seen in the right heart, notably in the right atrium wall. They are often different from the parenchyma around them and might be single or many [10,49]. Cardiovascular tuberculomas can be asymptomatic or present with pulmonary vein obstruction as a result of left atrial mass lesions, left ventricular aneurysm, right ventricular outflow tract obstruction, superior vena cava obstruction, coronary artery occlusion, ventricular dysfunction, ventricular rupture, aortic insufficiency or regurgitation, cardiac arrhythmia, complete heart block, or sudden cardiac death [50,51]. Nodular myocardial TB has been associated with the development of ventricular aneurysms [52]. True aneurysm development as a result of myocardial TB is very uncommon.

**Figure 5:**
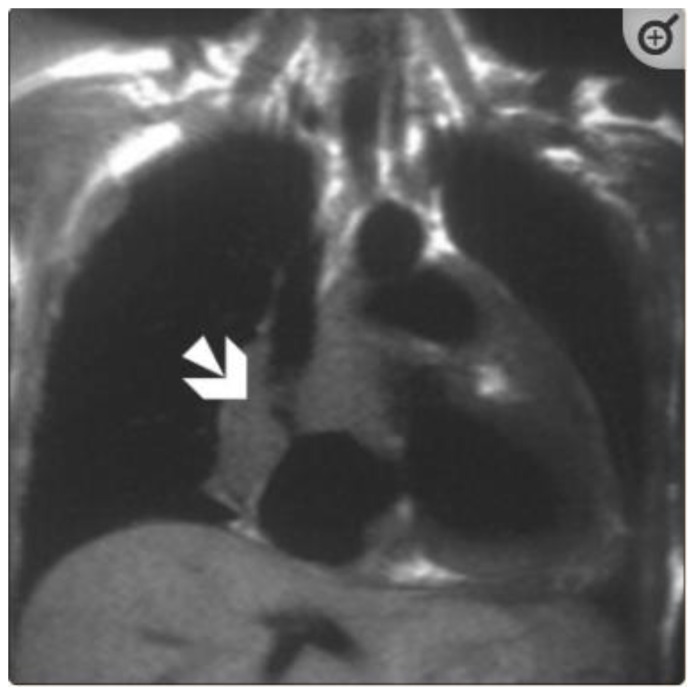
T2W dark-blood coronal MRI image shows diffuse myopericardial thickening. The thickening is hypointense on T2W images and is causing attenuation of the proximal SVC (arrow). The image was adapted from [53].

**Figure 6:**
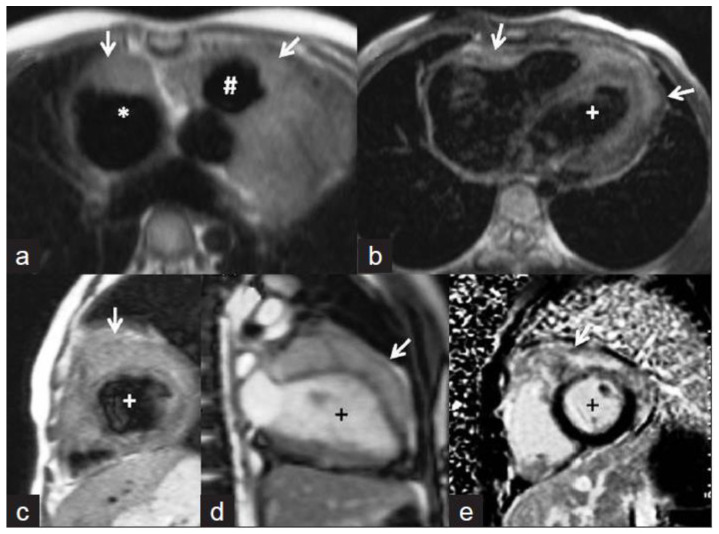
Cardiac magnetic resonance images. (**a**,**b**) Axial T1-weighted images showing isointense masses (arrows) along the anterior right atrium (*), right ventricular outflow tract (#), and along both ventricles (+ indicates left ventricle). (**c**) Short axis T2-weighted image showing that the lesions are mildly hyperintense. (**d**) Steady-state free precession image revealing the infiltrative nature of the lesion along the left ventricle. The delayed enhanced short-axis image (**e**) shows heterogeneous enhancement of the mass. The image was adapted from [47].

Cardiovascular magnetic resonance imaging is a relatively recent method that shows mild to moderate T2 shortening (Figure 5, This image was adapted from Dixit et al. [53] and 6, This image was adapted from Gulati et al. [47]) comparable to that seen in cerebral tuberculomas with tuberculous involvement [53]. A central isointense core belonging to central caseation, a hypointense rim relating to the fibrous capsule, and a thin hyperintense line about an inflammatory cellular infiltration may all be seen on T2W images [53]. Ring enhancement with conglomeration may be seen on post-gadolinium MRI (Figure 6, This image was adapted from Gulati et al. [47]). Conglomeration develops when a granulomatous lesion is present, which is uncommon in neoplasms [53].

### 5.5. CMR Feature Tracking

Myocarditis is a challenging diagnosis to establish, and there is no clear in vivo gold standard since a negative endomyocardial biopsy result does not exclude the diagnosis. In recent years, abnormal myocardial mechanics, mainly left ventricular global longitudinal strain (GLS), have been extensively studied to diagnose cardiac dysfunction in various cardiovascular diseases [54]. Additionally, myocardial strain abnormalities, ejection fraction, and other traditional risk factors are good predictors of bad outcomes in cardiovascular illnesses. Initially, most of this work was performed using customized CMR pulse sequences that needed substantial post-processing. More recently, the development of CMR “feature tracking” technology has enabled the quantification of GLS using standard cine acquisitions, obviating the need for particular pulse sequences. A single-center retrospective investigation examined the predictive effectiveness of feature tracking—derived GLS in 455 patients with a clinical presentation consistent with myocarditis [55]. Our CMR feature tracking results corroborated the experience mentioned above, with substantial decreases in GLS in the LV and the RV, with further reductions in the basal and mid RVCS (right ventricular circumferential strain).

### 5.6. Advantages of CMR with Echocardiography

Although echocardiography is the preferred imaging modality for the heart, CT and MR imaging are likely underutilized due to their capacity to increase lesion visibility and characterization. In addition, the imaging results are highly variable depending on the disease substrate. CMR is a promising technique for identifying pericardial inflammation and diagnosing and monitoring myocarditis [23,29,30,31]. CMR outperforms echocardiography in detecting tuberculous myocardial involvement, analogous to what has been reported in the Western European series on myopericarditis [28,56]. CMR also has several advantages over echocardiography identifying, characterizing, and assessing whole cardiac abnormalities. These include a high contrast resolution, an unlimited field of view, the ability to conduct multiplanar imaging and tissue characterization, multiparametric imaging, strain imaging, and a high degree of contrast variability. CMR is likely a more robust imaging modality in this context, as disseminated intracardiac tuberculomas have been demonstrated using gadolinium enhancement [12,28,47,57,58].

## 6. Role of Tc-99m Sestamibi and SPECT

Myocardial scintigraphy with Tc-99m pyrophosphate has also been helpful in myopericarditis, most notably TB myopericarditis when combined with Tc-99m sestamibi. In a case study, SPECT was more successful than CMR in locating a bioprosthetic valve abscess, and it may be helpful in tuberculosis research [8,59]. Gallium scintigraphy may be a more effective test for diagnosing TB myopericarditis than indium scintigraphy because it has a lower sensitivity for acute inflammation but a higher sensitivity for chronic inflammation [59]. Despite the widespread use of Tc-99m sestamibi in various cardiac diseases, its application in assessing myocardial viability remains disputed [60].

## 7. Role of Cardiac 18F-FDG PET

**Figure 7:**
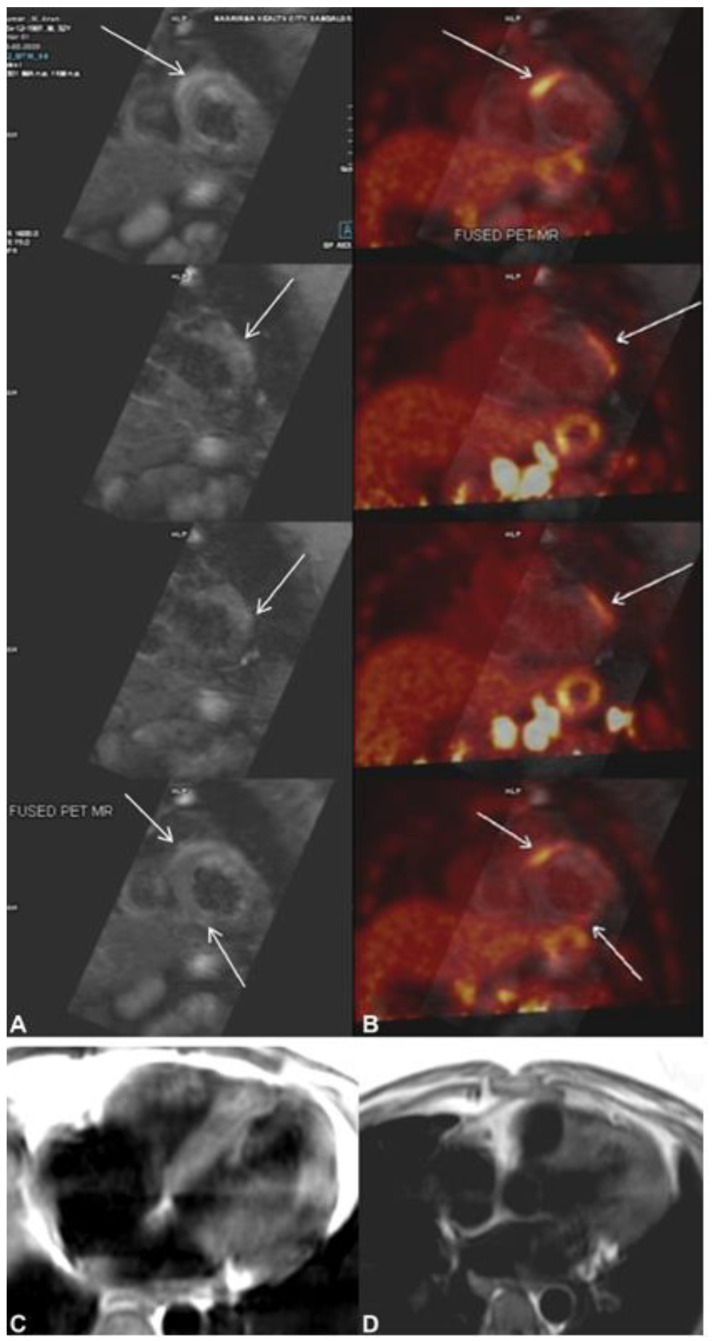
(**A**) Cardiac magnetic resonance (CMR) shows multifocal subepicardial to mid-myocardial linear enhancement along the right ventricular insertion site, mid-anterolateral, and inferior segments (arrows) with corresponding focal myocardial edema. (**B**) Fused cardiac positron emission tomography CMR shows patchy areas of increased ^18^F-flurodeoxyglycose (FDG) uptake in the apical to mid-anterolateral, mid-to-basal anteroseptal at the right ventricular insertion site, and mildly increased FDG uptake in the apical inferior segments of the left ventricular myocardium corresponding to the regions of myocardial enhancement seen on CMR. (**C**,**D**) T2 black blood image showing T2 hyperintense changes in the left ventricular myocardium. The image was adapted from [61].

Recently, cardiac TB was diagnosed using 18F-FDG PET. In one of their case reports, Sundaraiya et al. presented a case of cardiac tuberculosis that resembled sarcoidosis [61]. PET imaging revealed patchy regions of increased FDG uptake in the apical to mid anterolateral, mid to basal anteroseptal/right ventricular, and moderately increased FDG uptake in the apical inferior LV myocardium CMR findings (Figure 7, This image was adapted from Sundaraiya et al. [61]). On whole-body PET CT imaging, several hypermetabolic supra and infra diaphragmatic lymphadenopathy was detected, but no pulmonary lesion was detected. Inflammation of the necrotizing granulomatous type in the left para-aortic lymph node was consistent with tuberculosis [61]. Even though tuberculosis closely resembles sarcoidosis, FDG-PET is helpful. FDG-PET has several drawbacks compared to CMR, including non-inflammatory myocardial FDG uptake despite good FDG-PET scan preparation, false-positive results due to atrial fibrillation, or bundle branch block, which can affect regional glucose utilization, and radiation exposure [62].

## 8. Endomyocardial Biopsy

Over time, the importance of endomyocardial biopsy (EMB) in diagnosing pathogenic entities has evolved. In addition to myocarditis, EMB has a poor sensitivity rate of 25% for lymphocytic myocarditis and 35% for cardiac sarcoidosis [63]. The application of EMB to tuberculosis has not been discussed previously. Due to its low sensitivity but high specificity, EMB is the diagnostic method of choice for many disorders. Therefore, a high pre-test probability is necessary. In patients with a low pre-test likelihood, it is preferable to use other tests to rule out pathology. Among these may be scintigraphy (indium-111, gallium-67) [64,65]. The combination of CMR and EMB has a synergistic effect on myopericarditis diagnosis [66].

## 9. Advantage and Disadvantages of Imaging Modalities (CMR, CT, PET)

Transthoracic echocardiography, which combines structural and physiologic assessment, is a first-line technique for the examination of patients suspected of having or known to have myopericardial disease. However, the diagnostic accuracy is reduced in individuals with a poor acoustic window, and transthoracic echocardiography is limited in its ability to help identify localized effusions, assess pericardial thickness, and characterize tissue [67,68]. However, cardiac computed tomography (CT), magnetic resonance (MR), and positron emission tomography (PET) imaging are becoming increasingly popular for the study of this part of the heart in order to better define cardiac anatomy and function. In recent years, advancements in MR imaging technology and sequence design have boosted the diagnostic value of this modality for the evaluation of myopericardial disease in recent years. For example, fast MR sequences allow for real-time analysis of heart motion and inflow patterns during free-breathing, allowing for the finding of constrictive physiology [32,69,70]. Furthermore, delayed (or late) gadolinium-enhanced MR imaging is useful for diagnosing myopericardial inflammation and monitoring the effects of anti-inflammatory medication [33,71]. In constrictive pericarditis, MR tagging methods are useful in detecting both the fibrotic adherence of pericardial layers and cardiac involvement [72]. The intrapericardial contents may frequently be better appreciated using bright-blood dynamic cine MR imaging, such as the visibility of fibrinous threads or the presence of coagulated blood [73]. The standard imaging approach for detecting pericardial effusion is echocardiography. However, smaller effusions located accidentally in the posterior or inferior wall are undetectable by echocardiography [74]. On MR images, differentiation between pericardial thickening and effusion is typically straightforward.

The pericardium appears as a thin line of fibrous tissue on CT scans, but the visceral layer cannot be represented independently. The pericardial layers may be accurately shown via CT imaging, with thickness and content assessed. As a result, CT imaging can help physicians distinguish between basic pericardial effusions and inflammatory effusive pericarditis or malignant pericardial illnesses [75]. Furthermore, pericardial fluid may be characterized to some extent by assessing attenuation values on CT images. Using CT imaging, the increased pericardial X-ray absorption distinguishes pericardium from surrounding fatty tissue, such as mediastinal or epi-/pericardial fat. Notably, a distinct benefit of CT imaging is the capacity to identify pericardial calcification precisely and thoroughly, which can be crucial for distinguishing between constrictive pericarditis and the restrictive ventricular filling pattern [75,76,77]. Currently, CT is the best method for depicting even minute levels of pericardial calcium and for seeing issues such as the intramyocardial extension of the fibrocalcific process, which may impede the effectiveness of a pericardiectomy [75,76,77]. Preoperative CT may be effective for providing a thorough picture of both the degree of thickness and the presence and location of calcifications, so facilitating improved surgical planning and risk classification. However, pericardial calcifications are less prevalent now than in the past, which is likely due to the decline in TB rates and the rise in iatrogenic causes of constriction. Two recent investigations (74,90) indicated that 27% and 28% of patients with histologically verified constrictive pericarditis had pericardial calcifications. There have recently been reports on the use of CT in the identification of myocarditis; however, due to the intrinsic radiation, it cannot be used frequently for functional studies [78].

Unlike CT and MR imaging, which are based on anatomic structure and function, PET imaging reflects 18-fluorodeoxyglucose metabolism in vivo (FDG). PET imaging was employed in both strategies to correlate metabolic alterations.

The ability of FDG-PET to define inflammation and different stages of myocardial damage enables the early detection of cardiac infiltrative diseases such as sarcoidosis and TB and theoretically improves the test’s sensitivity at the price of specificity [61,79]. As a diagnostic tool, the increased sensitivity of FDG-PET is crucial for detecting illness at an earlier stage. Inflammatory processes involving FDG absorption are mild to moderate in the pericardium, and its use in detecting myopericardial TB is limited [61].

## 10. Remaining Challenges

Regardless of their financial circumstances, those with cardiovascular disease or those at high cardiovascular risk require early identification. The adoption of cardiac imaging technologies in poor nations faces several hurdles [80]. Some of the most powerful medical tools available to identify cardiovascular illness include diagnostic ultrasonography, angiography, computed tomography, and nuclear cardiology [81]. However, these services are still insufficiently available worldwide. While CMR is commonly employed in wealthy nations, its application in poor countries is either inconsistent or non-existent. According to the World Health Organization, despite the fact that x-rays and ultrasound can answer 70 to 80% of diagnostic issues, approximately two-thirds of the world’s population lacks access to basic diagnostic imaging. Ultrasound’s significance as a diagnostic cardiac modality is unrivaled in many ways. Portable echo has been shown to be useful in the evaluation of many different illnesses in impoverished nations, and it may also be used to examine heart function and hemodynamic state in patients such as ours [82].

## 11. Conclusions

Cardiac involvement is uncommon in tuberculosis, but it can lead to heart failure, constrictive pericarditis, or death; hence, early detection and management of these complications should be a top concern. Tuberculosis of the heart is a rare illness that can be lethal if left untreated. Expanding access to diagnostic tools, such as laboratory testing (such as the ADA or IFN-y) and imaging should be a priority. Even though echocardiography is still the standard for diagnosing myopericardial disorders, it remains a workhorse in underdeveloped nations due to a lack of access to other tests such as CT or CMR. However, techniques that provide improved tissue characterization are frequently required to support diagnosis and therapy. MRI may be used to obtain heart function and tissue morphology using a variety of acquisition protocols. CT, unlike MRI, involves the use of radiation. In addition to heart function and shape, CT can also show pericardial calcification. PET provides unique metabolic information that may be overlaid on CT data to help identify inflammatory processes or masses, such as neoplasms. These imaging modalities can be utilized to improve myopericardial TB diagnosis.

## Data Availability

Not applicable.

## Abbreviations

VT—ventricular tachycardia, CMR—cardiac magnetic resonance imaging, T2w STIR—T2-weighted short-tau inversion recovery, QFT—QuantiFERON-TB Gold, ECV—extracellular volume, TB—tuberculosis, HIV—human immunodeficiency virus, AL—anterolateral, LGE—late gadolinium enhancement, LVOT—left ventricular outflow tract, SPECT—Single-photon emission computed tomography and T1—spin-lattice relaxation time, 18F-FDG PET—18F-fluorodeoxyglucose Positron Emission Tomography, FDG—fluorodeoxyglucose, PET CT—Positron Emission Tomography Computed Tomography, Tc-99m—Technetium-99m, GLS—global longitudinal strain, RVCS—right ventricular circumferential strain, RV—right ventricle, DNA—deoxyribonucleic acid, PCR—polymerase chain reaction, ADA—adenosine deaminase, IFN-y—gamma interferon, HIV—human immunodeficiency virus, TB—tuberculosis.
